# A Cross-Sectional Study of Food Insecurity, Mediterranean Diet Adherence, and Gut-Derived Metabolites and Their Associations with Glycemic Control and Macrovascular Complications in Adults with Type 2 Diabetes

**DOI:** 10.3390/nu18162695

**Published:** 2026-08-18

**Authors:** Hatice Ozcaliskan Ilkay, Zuleyha Cihan Ozdamar Karaca

**Affiliations:** 1 Department of Nutrition and Dietetics, Faculty of Health Sciences, Erciyes University, 38260 Kayseri, Türkiye; 2 Department of Endocrinology, Erciyes University, 38030 Kayseri, Türkiye

**Keywords:** type 2 diabetes, food insecurity, diet, TMAO, L-carnitine, glycemic control, macrovascular complications

## Abstract

**Objective**: This study aimed to investigate the combined associations of food insecurity and Mediterranean diet adherence with glycemic control and to determine whether gut-derived metabolites differ according to food insecurity and macrovascular complication status in adults with type 2 diabetes mellitus (T2DM). **Methods**: In this cross-sectional study, food insecurity was assessed using the Food Insecurity Experience Scale, and Mediterranean diet adherence was evaluated with the 14-item Mediterranean Diet Adherence (MDA) Scale. Serum trimethylamine *N*-oxide (TMAO) and L-carnitine concentrations were quantified using enzyme-linked immunosorbent assay (ELISA). Multinomial logistic regression was used as the primary analysis of data-derived HbA1c tertiles, and separate binary logistic regression models were performed as sensitivity analyses. ANCOVA was used for adjusted comparisons of continuous outcomes. **Results**: In the primary multinomial analysis, none of the combined food insecurity–MDA patterns were significantly associated with the intermediate or highest HbA1c tertiles after multivariable adjustment. However, point estimates suggested higher odds of the intermediate HbA1c tertile among participants with food insecurity and high MDA adherence (AOR = 3.41, 95% CI: 0.76–15.25) and of the highest HbA1c tertile among those with food insecurity and low MDA adherence (AOR = 4.74, 95% CI: 0.66–34.16); these estimates were imprecise. Sensitivity analyses using separate binary logistic regression models yielded stronger associations, but these findings should also be interpreted cautiously. Participants with macrovascular complications exhibited significantly higher serum TMAO and L-carnitine concentrations (*p* = 0.026 and *p* = 0.039, respectively), whereas food-insecure participants had significantly lower concentrations of both metabolites (*p* = 0.009 and *p* = 0.018, respectively). Higher dietary fiber and plant protein intakes were cross-sectionally associated with lower TMAO concentrations, while L-carnitine concentrations were positively associated with dietary fiber intake and inversely associated with plant protein intake. **Conclusions**: Combined food insecurity–MDA patterns showed suggestive but imprecise differences in HbA1c category membership that require confirmation in larger prospective studies. Serum TMAO and L-carnitine concentrations differed according to food insecurity and macrovascular complication status and were associated with selected dietary components. These findings should be interpreted as exploratory cross-sectional associations rather than evidence of causal or mechanistic effects.

## 1. Introduction

Type 2 diabetes mellitus (T2DM) is a major global health challenge characterized by chronic hyperglycemia resulting from insulin resistance and/or inadequate insulin secretion, which leads to progressive metabolic dysregulation and a high burden of vascular complications [1,2]. Therefore, a proactive approach to the management of T2DM is crucial for preventing microvascular and macrovascular complications and alleviating the overall burden of mortality [3].

In this regard, the American Diabetes Association (ADA) particularly emphasizes the importance of adopting healthy and nutrient-dense dietary patterns—especially the Mediterranean diet—for achieving optimal metabolic control among adults with diabetes, as supported by consistent clinical evidence [2,4]. Adequate access to nutritious foods is thus a fundamental prerequisite for successful diabetes management, and food insecurity, defined by the ADA as “the unreliable availability of nutritious foods and the inability to obtain them in socially acceptable ways”, remains a significant barrier to achieving this goal [5]. Indeed, evidence consistently indicates that the prevalence of food insecurity is substantially higher among individuals with type 2 diabetes compared with national population estimates, underscoring its relevance as a social determinant of metabolic health [6,7]. Recent evidence has further highlighted the dual impact of food insecurity and diet quality on glycemic control among individuals with type 2 diabetes, indicating that the coexistence of food insecurity and poor diet quality is strongly associated with suboptimal glycemic control [8].

Gut-derived metabolites such as trimethylamine *N*-oxide (TMAO) and its dietary precursor L-carnitine have recently attracted considerable attention because of their reported associations with cardiometabolic health [9,10,11,12,13]. Specifically, higher circulating TMAO concentrations have been associated with atherosclerosis, metabolic syndrome, T2DM, and cardiovascular and renal complications of T2DM [9,14]. Experimental studies suggest that TMAO may influence cardiometabolic health through several interconnected mechanisms, including impaired reverse cholesterol transport, endothelial dysfunction, oxidative stress, chronic low-grade inflammation, and enhanced platelet activation, all of which may promote atherosclerosis and vascular injury [12,14,15]. TMAO has also been implicated in disturbances of glucose and lipid metabolism, insulin resistance, and tissue fibrosis, potentially contributing to the progression of type 2 diabetes mellitus (T2DM) and its cardiovascular and renal complications [14,15]. However, although these mechanisms are biologically plausible, current human evidence remains largely observational and heterogeneous, and the causal role of TMAO in cardiometabolic disease has not yet been established. Similarly, although L-carnitine plays an essential role in fatty acid oxidation, its microbial conversion to TMAO has been implicated in pathways associated with atherosclerosis and cardiovascular disease [13].

Growing evidence suggests that circulating TMAO concentrations are associated with dietary patterns and cardiometabolic risk, although the underlying biological mechanisms remain incompletely understood. For example, plant-based and fiber-rich diets, such as the Mediterranean diet, have been associated with lower circulating TMAO concentrations, whereas animal-based and high-meat diets have been associated with higher concentrations [16]. These dietary patterns may reduce circulating TMAO concentrations by limiting the availability of dietary TMA precursors, including L-carnitine and choline, while promoting a gut microbial composition that favors beneficial metabolite production over trimethylamine generation [17,18,19]. In addition, dietary fiber supports short-chain fatty acid production, intestinal barrier integrity, and metabolic homeostasis, thereby modulating gut microbiota–host interactions [18].

Despite extensive research on the metabolic implications of these gut-derived metabolites, little is known about how they are associated with specific social determinants and dietary components—including food insecurity, dietary fiber intake, and plant-based protein consumption. Moreover, despite substantial research on dietary management in type 2 diabetes mellitus (T2DM), limited evidence exists regarding how food insecurity and diet quality are jointly associated with metabolic profiles through gut-derived pathways. These research gaps are clinically relevant, as both social adversity and alterations in microbial metabolism may contribute to poor glycemic control and increased cardiovascular risk.

Thus, given the growing recognition of trimethylamine *N*-oxide (TMAO) and L-carnitine as gut-derived metabolites associated with diet and vascular health, in this study, it is hypothesized that food insecurity and Mediterranean diet adherence would be jointly associated with glycemic control, and that circulating TMAO and L-carnitine concentrations, together with their dietary correlates, would vary according to food insecurity and macrovascular complication status in adults with T2DM.

## 2. Materials and Methods

### 2.1. Study Design and Data Collection

This cross-sectional study recruited 120 adults with type 2 diabetes mellitus (T2DM) from the Endocrinology and Metabolism Outpatient Clinic of Erciyes University Hospital between November 2024 and March 2025 using a consecutive sampling approach. Eligible participants were 18–75 years of age, had a confirmed T2DM diagnosis with a duration of at least one year, and had not undergone any changes in their medical treatment during the preceding three months. Individuals were excluded if they were pregnant or lactating; had cognitive impairment, an autoimmune disease, a systemic infection, impaired renal function, a terminal illness, or malignancy; or had used antibiotics or probiotics regularly within the past three months. Data were collected through face-to-face interviews conducted by trained intern dietitians using a structured questionnaire administered to participants who met the inclusion criteria and had provided written informed consent. The presence of diabetes-related chronic complications (macrovascular and microvascular) was evaluated by endocrinologists during routine clinical examinations and/or verified through patients’ medical records. Macrovascular complications were defined as the presence of clinically diagnosed coronary artery disease (including myocardial infarction and congestive heart failure), cerebrovascular disease (including stroke and cerebral ischemia), and peripheral vascular disease (including intermittent claudication, critical limb ischemia, and lower-extremity amputation) based on the participants’ medical records and physician diagnosis. Dietary habits, food consumption frequency, and a one-day 24-h dietary recall were also obtained. Additionally, validated tools such as the Food Insecurity Experience Scale and the Mediterranean Diet Adherence Screener were administered, and physical activity levels were evaluated using the International Physical Activity Questionnaire–Short Form (IPAQ-SF).

Sample size was calculated using G*Power 3.1 (f = 0.35, power = 80%, α = 0.05), indicating a minimum of 100 participants. To ensure adequate statistical power and account for potential data loss, 120 participants were ultimately included.

### 2.2. Food Insecurity Experience Scale (FIES)

The Food Insecurity Experience Scale (FIES), designed by the FAO, enables reliable and cross-culturally comparable assessment of food insecurity severity [20,21,22]. In the Turkish context, food insecurity was measured using the FIES within the framework of the Turkey Nutrition and Health Survey-2017 (TBSA-2017) [23], and a Turkish validity and reliability study of the scale was conducted by Kılınç et al. [24]. The scale comprises eight questions with “yes” or “no” response options. In this study, participants who answered “no” to all questions were classified as food-secure; those who answered “yes” to 1–3 items were categorized as having mild food insecurity; those who answered “yes” to 4–7 items were considered to have moderate food insecurity; and those who answered “yes” to all eight items were classified as experiencing severe food insecurity.

### 2.3. Mediterranean Diet Adherence (MDA) Scale

Compliance with the Mediterranean diet was determined using the 14-item Mediterranean Diet Adherence Scale, developed by Martínez-González et al. [25] and later adapted and validated for use in Turkey by Bekar et al. [26]. The MDA Scale comprises two items targeting overall dietary behaviors and twelve items measuring how often specific Mediterranean diet food groups are consumed. Item scores (0 or 1) are summed to yield a total score of 0–14, where higher values represent greater compliance with the Mediterranean diet.

### 2.4. International Physical Activity Questionnaire (IPAQ)–Short Form

Physical activity was assessed using the International Physical Activity Questionnaire (IPAQ), a widely utilized self-report instrument that measures physical activity performed within the last seven days across multiple activity domains, including occupational, transport, domestic, and leisure-time activities [27]. The Turkish adaptation, validated by Sağlam et al. [28], has been demonstrated to possess high reliability and validity in adult Turkish populations. In this study, physical activity levels are reported as MET-minutes per week.

### 2.5. Anthropometric and Body Composition Measurements

Anthropometric data—including height, body weight, waist circumference (WC), hip circumference, and neck circumference—were collected once by trained dietetic interns following standardized procedures [29,30]. Body mass index was computed based on measured body weight and height values [29,30].

### 2.6. Measurement of Serum Metabolites and Biochemical Parameters

For the analysis of serum trimethylamine *N*-oxide (TMAO) and L-carnitine levels, venous blood samples were collected from patients after fasting for at least 8 h into BD Vacutainer^®^ 5 mL Serum Separation Tubes (Beckton, Dickinson and Company, Franklin Lakes, NJ, USA) and allowed to clot at room temperature for 20 min. After clot formation, the samples were centrifuged, and the separated serum was aliquoted and stored at −80 °C until analysis. Once all samples had been collected, serum trimethylamine *N*-oxide (TMAO) and serum L-carnitine concentrations were determined using commercially available human ELISA kits (TMAO: Bioassay Technology Laboratory [BT LAB], Shanghai, China, Catalog No. E4733Hu; L-carnitine: MyBioSource, San Diego, CA, USA, Catalog No. MBS701318) according to the manufacturers’ instructions. The TMAO assay had a standard curve range of 0.2–60 ng/mL, and the L-carnitine assay had a detection range of 62.5–4000 pg/mL and an analytical sensitivity of 15.6 pg/mL. According to the manufacturers, the intra-assay and inter-assay coefficients of variation are <8% and <10%, respectively. Absorbance was measured at 450 nm using a BioTek ELx800 microplate reader (BioTek Instruments, Winooski, VT, USA) [31]. One sample was excluded due to technical limitations.

Additionally, routine biochemical parameters retrieved from clinical records were analyzed using standardized laboratory procedures. Fasting plasma glucose was measured by an enzymatic spectrophotometric method (Roche Diagnostics, Mannheim, Germany, Cobas 6000 analyzer). Glycated hemoglobin (HbA1c) was assessed using a turbidimetric inhibition immunoassay (Roche Diagnostics, Tina-quant HbA1c Gen.3). Lipid profile components—including total cholesterol, high-density lipoprotein cholesterol (HDL-C), low-density lipoprotein cholesterol (LDL-C), and triglycerides—were determined via enzymatic spectrophotometric assays (Roche Diagnostics, Cobas 6000 analyzer). All biochemical analyses were conducted in the Biochemistry Laboratory of Erciyes University Hospital and the GENKÖK Biochemistry Laboratory (Genome and Stem Cell Center, Erciyes University).

### 2.7. Statistical Analysis

Statistical analyses were performed using IBM SPSS Statistics, version 23.0 (Armonk, NY, USA). The normality of continuous variables was assessed using histograms, probability plots, and the Kolmogorov–Smirnov test. For the baseline analyses, continuous variables are summarized as mean ± standard deviation, with median values reported in parentheses. Group differences in normally distributed continuous variables were examined using one-way analysis of variance (ANOVA), while the Kruskal–Wallis test was applied for non-normally distributed variables. Categorical variables are presented as frequencies and percentages, and comparisons across groups were evaluated using the chi-square test. In the analyses adjusted for covariates, descriptive statistics for continuous variables are reported either as crude means with standard deviations or as adjusted means with standard errors, whereas categorical variables are summarized using frequency distributions. Crude means reflect unadjusted group values and are provided for descriptive purposes. Adjusted means were derived using analysis of covariance (ANCOVA), with relevant confounders statistically controlled. ANCOVA models were adjusted for age, duration of type 2 diabetes mellitus, gender, educational level, marital status, antidiabetic medication use, insulin therapy, and total physical activity level. For analyses involving diet-related variables, total energy intake was included as an additional covariate to account for variations in overall dietary consumption. A multinomial logistic regression model was fitted as the primary analysis to examine associations between combined food insecurity–Mediterranean diet adherence patterns and the three mutually exclusive HbA1c categories, using HbA1c < 6.62% as the reference outcome and the food-secure/high Mediterranean diet adherence group (FI−/MDA↑) as the reference exposure. The model was adjusted for age, duration of type 2 diabetes mellitus, gender, body mass index, insulin therapy, oral antidiabetic medication use, physical activity, and total energy intake. HbA1c was categorized into tertiles based on its distribution in the study population, resulting in three equally sized groups (n = 40 per tertile): <6.62%, 6.62–8.22%, and >8.22%. These data-driven categories were used to obtain balanced subgroup sizes for regression analyses and do not represent established clinical thresholds.

In the graphical representations, adjusted means and their corresponding standard errors derived from the ANCOVA models are presented. A two-sided *p*-value < 0.05 was considered statistically significant.

Prior to fitting the adjusted regression models, multicollinearity among the covariates was assessed using variance inflation factors (VIFs). All VIF values ranged from 1.08 to 1.47, indicating no evidence of problematic multicollinearity. Because of the modest sample size, adjustment was restricted to clinically relevant covariates identified a priori (age, gender, duration of diabetes, body mass index, insulin use, oral antidiabetic medication, physical activity, and energy intake). As a sensitivity analysis, three separate multivariable binary logistic regression models were fitted in which each HbA1c category was compared with the remaining two categories. For these binary sensitivity models, discrimination and calibration were evaluated using the area under the receiver operating characteristic curve (AUC) and the Hosmer–Lemeshow goodness-of-fit test, respectively.

The ANCOVAs were hypothesis-driven and focused on a predefined set of dietary variables selected a priori based on their biological relevance to gut-derived metabolites. Therefore, nominal *p*-values are reported. To facilitate interpretation beyond statistical significance, partial eta-squared (partial η^2^) values are presented as measures of effect size for all ANCOVAs, whereas adjusted odds ratios with 95% confidence intervals are reported for logistic regression models.

## 3. Results

The sociodemographic, lifestyle, and cardiometabolic characteristics of participants with type 2 diabetes, stratified by combined food insecurity and Mediterranean diet adherence categories, are presented in Table 1. As can be seen, when food insecurity and Mediterranean diet adherence are combined into a four-level variable, 30.0% (n = 36) of participants are classified as food-secure with low Mediterranean diet adherence (FI−/MDA↓), 13.3% (n = 16) as food-insecure with low adherence (FI+/MDA↓), 20.8% as food-secure with high adherence (FI−/MDA↑), and 35.8% as food-insecure with high adherence (FI+/MDA↑).

**Table 1:** Sociodemographic, lifestyle, and cardiometabolic characteristics of adults with type 2 diabetes across combined food insecurity and Mediterranean diet adherence categories.

	Overall (n = 120)	FI−/MDA↓ (n = 36)	FI+/MDA↓ (n = 16)	FI−/MDA↑ (n = 25)	FI+/MDA↑ (n = 43)	*p*
Age (years)	58.6 ± 10.0 (59.0)	59.6 ± 9.4	59.4 ± 8.0	58.2 ± 12.3	57.6 ± 9.8	0.797
Duration of T2DM (years)	11.6 ± 6.5 (10.5)	11.8 ± 7.0	12.0 ± 5.7	11.1 ± 6.7	11.5 ± 6.5	0.954
Gender, n (%)						0.210
Men	19 (15.8)	9 (25.0)	1 (6.3)	2 (8.0)	7 (16.3)	
Women	101 (84.2)	27 (75.0)	15 (93.7)	23 (92.0)	36 (83.7)	
Current smoker [n (%)]	18 (15.0)	7 (19.4)	3 (18.8)	4 (16.0)	4 (9.3)	0.604
Physical activity level (MET-min/week)	418.1 ± 547.1 (219.2)	624.5 ± 744.1	311.7 ± 291.7	410.9 ± 464.9	289.1 ± 418.2	0.057
Educational level, n (%)						**0.027**
Primary or below	74 (61.7)	19 (52.8)	15 (93.8)	13 (52.0)	27 (62.8)	
Above primary	46 (38.3)	17 (47.2)	1 (6.3)	12 (48.0)	16 (37.2)	
Marital status, n (%)						0.101
Married	99 (82.5)	29 (80.6)	11 (68.8)	19 (76.0)	40 (93.0)	
Single	21 (17.5)	7 (19.4)	5 (31.3)	6 (24.0)	3 (7.0)	
Income level, n (%)						0.087
Insufficient	29 (24.2)	8 (22.2)	7 (43.8)	3 (12.0)	11 (25.6)	
Adequate	76 (63.3)	20 (55.6)	9 (56.3)	18 (72.0)	29 (67.4)	
Surplus	15 (12.5)	8 (22.2)	0 (0.0)	4 (16.0)	3 (7.0)	
Body mass index (kg/m^2^)	32.9 ± 6.1 (32.0)	33.4 ± 6.3	31.6 ± 6.3	31.7 ± 5.0	33.6 ± 6.5	0.542
<30 kg/m^2^ [n (%)]	43 (35.8)	10 (27.8)	7 (43.8)	11 (44.0)	15 (34.9)	
≥30 kg/m^2^ [n (%)]	77 (64.2)	26 (72.2)	9 (56.3)	14 (56.0)	28 (65.1)	
Macrovascular complications, n (%)						0.381
Absent	82 (68.3)	21 (58.3)	11 (68.8)	17 (68.0)	33 (76.7)	
≥1 present	38 (31.7)	15 (41.7)	5 (31.3)	8 (32.0)	10 (23.3)	
Oral antidiabetic therapy, n (%)	112 (93.3)	34 (94.4)	15 (93.8)	24 (96.0)	39 (90.7)	0.839
Insulin therapy, n (%)	55 (45.8)	14 (38.9)	7 (43.8)	11 (44.0)	23 (53.5)	0.620
HbA1c (%)	7.8 ± 1.7	7.5 ± 1.6	8.2 ± 2.1	7.9 ± 1.8	8.0 ± 1.7	0.307
TMAO (ng/mL)	1.47 ± 0.48 (1.29)	1.56 ± 0.48	1.28 ± 0.28	1.62 ± 0.53	1.37 ± 0.47	**0.013**
L-carnitine (ng/mL)	1.32 ± 0.50 (1.13)	1.38 ± 0.56	1.09 ± 0.23	1.52 ± 0.57	1.24 ± 0.44	0.051
MDA score	6.44 ± 2.41 (6.0)	4.06 ± 0.92	4.56 ± 0.89	7.64 ± 1.63	8.44 ± 1.65	**<0.001**
Food insecurity status, n (%)						**<0.001**
Food-secure	20 (16.7)	9 (25.0)	0 (0.0)	11 (44.0)	0 (0.0)	
Mild food insecurity	41 (34.2)	27 (75.0)	0 (0.0)	14 (56.0)	0 (0.0)	
Moderate/severe food insecurity	59 (49.2)	0 (0.0)	16 (100.0)	0 (0.0)	43 (100.0)	

Boldface *p*-values indicate statistical significance at *p* < 0.05. Data are presented as mean ± standard deviation and median (in parentheses) for continuous variables and as numbers (percentages) for categorical variables. **FI:** food insecurity status (− secure, + insecure); **MDA:** adherence to Mediterranean diet (↓ low adherence, ↑ high adherence). FI (−) represents no or mild food insecurity, whereas FI (+) represents moderate or severe food insecurity. Similarly, MDA (↓) represents low adherence, whereas MDA (↑) represents moderate or high MDA adherence. The grouping variable combining food insecurity and Mediterranean diet adherence (FI−/MDA↓, FI+/MDA↓, FI−/MDA↑, FI+/MDA↑) was created to compare the cumulative characteristics of participants according to their food security and dietary adherence status, as well as to enable the advanced analyses presented in Table 2. The “Food insecurity status” variable in this table represents the distribution of participants across the original Food Insecurity Experience Scale (FIES) categories (food-secure, mild, and moderate/severe). TMAO: trimethylamine *N*-oxide.

**Table 2:** Adjusted odds ratios (AORs) for combined food insecurity–Mediterranean diet adherence patterns across HbA1c tertiles from the primary multinomial logistic regression analysis among patients with T2DM. Reference outcome: HbA1c < 6.62%; reference exposure: food-secure × high Mediterranean diet adherence (FI−/MDA↑).

Pattern of Food Insecurity × Mediterranean Diet Adherence	HbA1c 6.62–8.22 vs. <6.62 AOR (95% CI)	*p*	HbA1c > 8.22 vs. <6.62 AOR (95% CI)	*p*
**FI−/MDA↓**	0.67 (0.17–2.71)	0.579	0.30 (0.06–1.50)	0.140
**FI+/MDA↓**	1.69 (0.24–11.67)	0.597	4.74 (0.66–34.16)	0.123
**FI+/MDA↑**	3.41 (0.76–15.25)	0.108	1.26 (0.21–7.52)	0.799
**FI−/MDA↑**	Reference	—	Reference	—

FI: food insecurity status (− secure, + insecure); MDA: adherence to Mediterranean diet (↓ low adherence, ↑ high adherence). Reference outcome: HbA1c <6.62%; reference exposure: FI−/MDA↑. AORs are adjusted for age, duration of type 2 diabetes mellitus, gender, body mass index, total physical activity level, oral antidiabetic medication use, insulin therapy, and total energy intake. HbA1c categories were derived from tertiles of the study population and do not represent established clinical thresholds.

In terms of demographics, a total of 120 adults with T2DM participated in the study (84.2% women; mean age 58.6 ± 10.0 years). The median duration of diabetes was 10.5 years, and the mean BMI was 32.9 ± 6.1 kg/m^2^, with 64.2% of participants being classified as obese (BMI ≥ 30 kg/m^2^). Nearly one-third of participants (31.7%) had at least one macrovascular complication. Most participants were married (82.5%), had completed at least a primary school education (61.7%), and reported adequate financial status (63.3%). The majority were receiving oral antidiabetic medications (93.3%), and 45.8% were also using insulin. The mean HbA1c level was 7.8 ± 1.7%. Mean serum TMAO and L-carnitine concentrations were 1.47 ± 0.48 ng/mL and 1.32 ± 0.50 ng/mL, respectively. The median Mediterranean Diet Adherence (MDA) score was 6.0. Food insecurity was highly prevalent, with 49.2% experiencing moderate-to-severe food insecurity and only 16.7% being fully food-secure. Only educational level, TMAO and L-carnitine concentrations, and—as expected—the MDA score and food insecurity status differed across the groups defined by food insecurity–dietary adherence categories.

The grouping variable combining food insecurity and Mediterranean diet adherence (FI−/MDA↓, FI+/MDA↓, FI−/MDA↑, FI+/MDA↑) was created to compare the cumulative characteristics of participants according to their food security and dietary adherence status, as well as to enable the advanced analyses presented in Table 2. The “Food insecurity status” variable in this table represents the distribution of participants across the original Food Insecurity Experience Scale (FIES) categories (food-secure, mild, and moderate/severe).

Table 2 presents the adjusted odds ratios (AORs) from the primary multinomial logistic regression analysis examining associations between combined food insecurity and Mediterranean diet adherence (MDA) patterns and the data-derived HbA1c tertiles. The lowest HbA1c tertile (<6.62%) was used as the reference outcome, and the food-secure/high-MDA group (FI−/MDA↑) served as the reference exposure. After multivariable adjustment, none of the combined food insecurity–MDA patterns were significantly associated with membership in the intermediate or highest HbA1c tertiles. Nevertheless, the point estimates suggested higher odds of the intermediate HbA1c tertile among participants with food insecurity and high MDA adherence (FI+/MDA↑; AOR = 3.41, 95% CI: 0.76–15.25; *p* = 0.108) and higher odds of the highest HbA1c tertile among participants with food insecurity and low MDA adherence (FI+/MDA↓; AOR = 4.74, 95% CI: 0.66–34.16; *p* = 0.123). The confidence intervals were wide, indicating limited precision, and these subgroup-specific estimates should therefore be interpreted cautiously as exploratory findings.

In sensitivity analyses using three separate adjusted binary logistic regression models, stronger associations were observed for some subgroup comparisons, including food insecurity combined with high MDA adherence in the intermediate and highest HbA1c categories (Supplementary Table S1). However, because this alternative modeling strategy compares each HbA1c category with the other two categories combined, these findings are considered supportive sensitivity results rather than the primary evidence. Diagnostics for the binary sensitivity models showed no problematic multicollinearity (all VIFs 1.08–1.47), with good discrimination for the lowest and highest HbA1c categories and acceptable discrimination for the intermediate category. Given the differences in statistical significance across modeling approaches and the wide confidence intervals, the HbA1c findings are interpreted as hypothesis-generating and require confirmation in larger prospective studies.

Adjusted mean values of macronutrient intake according to serum TMAO concentration are presented in Table 3. Here, participants are divided into two groups based on median serum TMAO concentration, and, after adjustment for age, duration of diabetes, gender, antidiabetic medication use, insulin therapy, total physical activity level, and energy intake, several significant differences can be observed: Individuals with lower TMAO concentrations (<1.29 ng/mL) had significantly higher intakes of total dietary fiber (15.21 ± 0.69 vs. 13.95 ± 0.67 g/day; *p* = 0.004), soluble fiber (4.74 ± 0.27 vs. 4.55 ± 0.27 g/day; *p* = 0.029), and insoluble fiber (10.21 ± 0.51 vs. 9.01 ± 0.50 g/day; *p* = 0.004) than those with higher TMAO levels. Moreover, plant protein intake was slightly but significantly greater among participants with lower TMAO levels (21.32 ± 0.99 vs. 20.81 ± 0.97 g/day; *p* = 0.040). No significant differences were observed for carbohydrate, total protein, total fat, or specific fatty acid intakes, or for cholesterol or MDA score (*p* > 0.05 for all). Taken together, these findings indicate that higher dietary fiber and plant protein intakes were cross-sectionally associated with lower circulating TMAO concentrations among adults with T2DM, supporting further investigation of dietary factors as potential correlates of gut-derived metabolite profiles.

Adjusted mean values of macronutrient intake according to serum L-carnitine concentration are shown in Table 4. Participants are classified into two groups based on median serum L-carnitine concentration, and, after adjustment for age, duration of diabetes, gender, antidiabetic medication use, insulin therapy, total physical activity level, and energy intake, significant differences can be observed: Participants with higher L-carnitine concentrations (≥1.14) had greater total dietary fiber intake (14.88 ± 0.69 vs. 14.27 ± 0.67 g/day, *p* = 0.008), including intake of soluble fiber (4.86 ± 0.27 vs. 4.44 ± 0.26 g/day, *p* = 0.018) and insoluble fiber (9.72 ± 0.51 vs. 9.48 ± 0.50 g/day, *p* = 0.013), compared with those with lower L-carnitine levels. In contrast, plant protein intake was slightly lower among participants in the higher-L-carnitine group (20.65 ± 0.99 vs. 21.45 ± 0.96 g/day, *p* = 0.037). No significant differences were detected for carbohydrate, total protein, total fat, or specific fatty acid intakes, or for cholesterol or MDA score (*p* > 0.05 for all). Overall, these results indicate that higher circulating L-carnitine concentrations were positively associated with total, soluble, and insoluble fiber intake, whereas plant protein intake had an inverse association among adults with T2DM.

**Figure 1:**
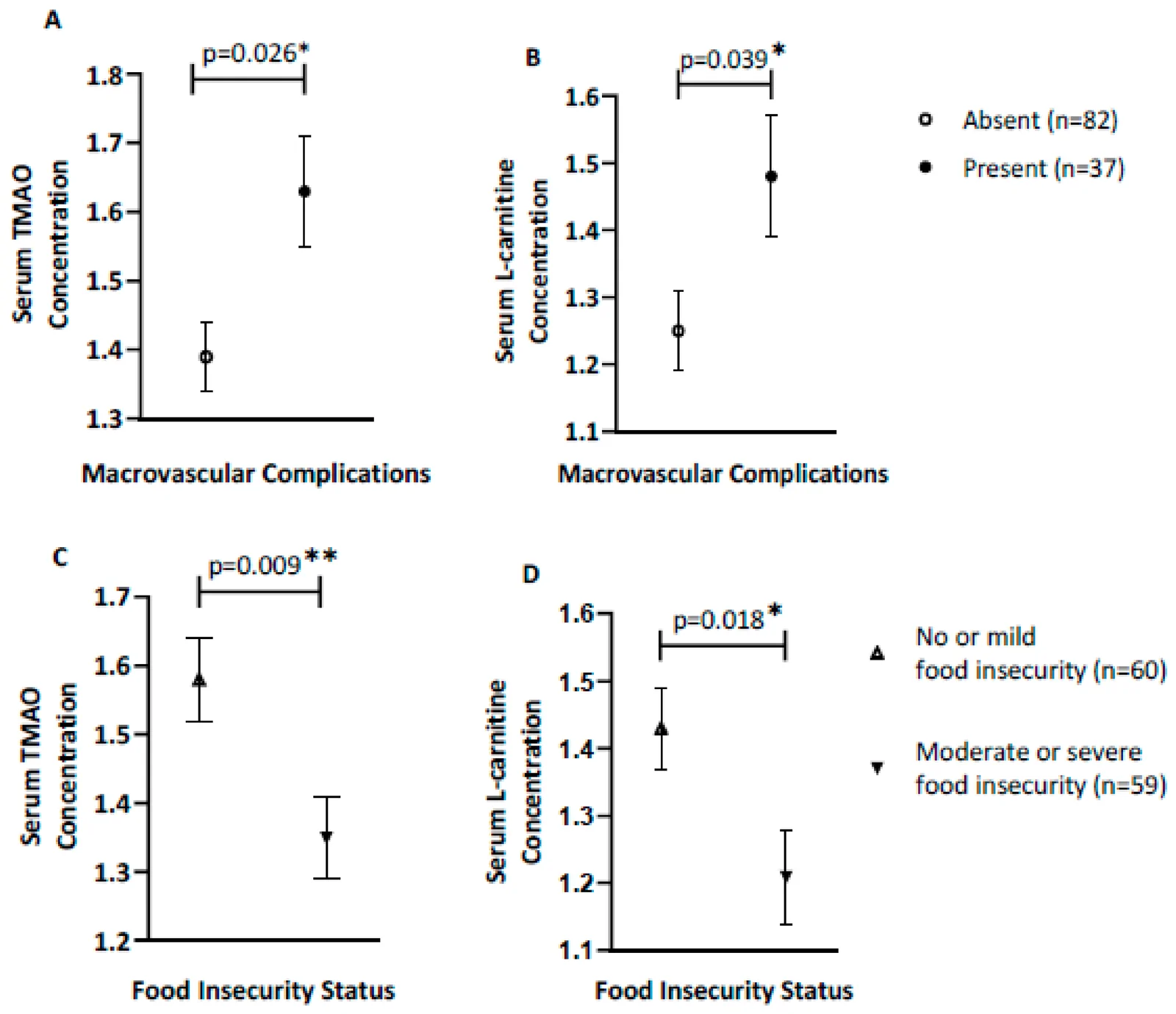
Adjusted mean (±standard error) values of serum TMAO and L-carnitine concentrations according to macrovascular complications and food insecurity status among patients with T2DM. (**A**) Serum TMAO concentrations according to macrovascular complication status, (**B**) serum L-carnitine concentrations according to macrovascular complication status, (**C**) serum TMAO concentrations according to food insecurity status, (**D**) serum L-carnitine concentrations according to food insecurity status. Exact adjusted means, adjusted mean differences, 95% confidence intervals, effect sizes (partial η^2^), and exact *p*-values corresponding to Figure 1 are provided in Supplementary Table S4. Values are adjusted for age, duration of type 2 diabetes mellitus, gender, antidiabetic medication use, insulin therapy, total physical activity level, and body mass index using ANCOVA. Open and closed circles represent the absence and presence of macrovascular complications, respectively, and upward and downward triangles represent no/mild and moderate/severe food insecurity, respectively. Asterisks indicate statistical significance (* *p* < 0.05, ** *p* < 0.01). The figure was created using GraphPad Prism version 10 (GraphPad Software, San Diego, CA, USA). TMAO: trimethylamine *N*-oxide. Serum TMAO and L-carnitine concentrations are expressed in ng/mL.

Adjusted mean (±standard error) serum concentrations of TMAO and L-carnitine, estimated from ANCOVA models adjusted for age, duration of diabetes, gender, antidiabetic medication use, insulin therapy, physical activity, and body mass index, are presented in Figure 1. Patients with macrovascular complications had significantly higher serum TMAO (1.63 ± 0.08, 1.39 ± 0.05; *p* < 0.05, respectively) and L-carnitine (1.48 ± 0.09, 1.25 ± 0.06; *p* < 0.05, respectively) concentrations compared with those without complications (Figure 1A,B). On the other hand, participants experiencing moderate or severe food insecurity exhibited markedly lower serum TMAO (1.35 ± 0.06, 1.58 ± 0.06; *p* < 0.01, respectively) and L-carnitine (1.20 ± 0.40, 1.44 ± 0.56; *p* < 0.05, respectively) levels than those reporting no or mild food insecurity (Figure 1C,D). Overall, higher serum TMAO and L-carnitine levels were linked to the presence of macrovascular complications, whereas lower levels were observed in participants with greater food insecurity.

## 4. Discussion

This study provides novel insights into the cross-sectional relationships among food insecurity, dietary quality, gut-derived metabolites, glycemic control, and macrovascular complications in adults with T2DM. In the primary multinomial analysis, the combined food insecurity–Mediterranean diet adherence patterns were not significantly associated with HbA1c tertile membership after multivariable adjustment, although some point estimates suggested higher odds of less favorable HbA1c categories among food-insecure subgroups. These estimates were imprecise and should be considered exploratory. Individuals with macrovascular complications had significantly higher serum concentrations of TMAO and L-carnitine, whereas those experiencing greater food insecurity showed lower levels of these metabolites. Nutritional analyses also identified cross-sectional associations of circulating TMAO and L-carnitine concentrations with selected dietary components, including dietary fiber and plant protein. Collectively, these findings highlight a potentially important interplay between food insecurity, dietary factors, and gut-derived metabolites in T2DM while underscoring the need for prospective studies to clarify their clinical and mechanistic relevance.

Emerging evidence indicates that, among adults with diabetes, food insecurity is associated with poorer glycemic control through multiple social, behavioral, and nutritional pathways [32]. In this context, the role of specific dietary patterns in supporting glycemic stability remains relevant. In a recent network meta-analysis of ten dietary interventions in adults with type 2 diabetes, the Mediterranean diet ranked among the top-performing patterns, showing the second-highest SUCRA for HbA1c (81.1%) and the third-highest for fasting glucose (76.3%) and body weight reduction (73.4%) [2]. Casagrande et al. [8] analyzed data from 2075 adults with diagnosed diabetes in the U.S. (NHANES 2013–2018) and found that individuals experiencing food insecurity combined with low diet quality were more likely to have elevated HbA1c levels (≥7.0%: AOR = 1.85 [1.23–2.80]; ≥8.0%: AOR = 1.79 [1.04–3.08]) than those who were food-secure and had high diet quality. In the present study, the primary multinomial model did not identify statistically significant differences in HbA1c tertile membership across the combined food insecurity–MDA patterns. In separate binary logistic regression models conducted as sensitivity analyses, food insecurity combined with high MDA adherence was significantly associated with greater odds of belonging to the intermediate (AOR = 5.18, 95% CI: 1.40–19.2) and highest (AOR = 4.32, 95% CI: 1.39–13.4) HbA1c categories compared with food security combined with high MDA adherence (Supplementary Table S1). Given the wide confidence intervals and inconsistency in statistical significance across modeling approaches, these observations should be regarded as hypothesis-generating rather than definitive evidence of an independent effect of food insecurity or of attenuation of the benefits of higher diet quality.

The pattern of estimates across food insecurity and MDA categories warrants cautious consideration. Several biological, behavioral, and social explanations could be proposed, but these remain speculative given the cross-sectional design. Residual confounding from unmeasured factors, including socioeconomic circumstances, healthcare access, medication adherence, psychological stress, or other lifestyle behaviors, may have influenced the observed associations despite adjustment for clinically relevant covariates. Reverse causation is also possible, whereby individuals with poorer glycemic control may have adopted healthier dietary habits following medical advice after diabetes diagnosis. Furthermore, Mediterranean diet adherence was assessed using a self-reported questionnaire and may be subject to measurement error or recall bias. The relatively small subgroup sizes also resulted in wide confidence intervals and limited statistical precision. Accordingly, the HbA1c findings should be considered exploratory and require confirmation in larger prospective studies.

Given the modest sample size, subgroup-specific estimates should be interpreted with appropriate caution. To minimize the risk of model overfitting, adjustment was restricted to clinically relevant covariates selected a priori based on biological plausibility and previous evidence. Multicollinearity was not problematic (all VIFs 1.08–1.47). The primary multinomial model appropriately accounted for the mutually exclusive three-level HbA1c outcome, whereas the separate binary models were retained as sensitivity analyses. Because the magnitude and statistical significance of some estimates differed across these modeling approaches, and confidence intervals were wide, the results do not provide definitive evidence of independent subgroup effects and should be confirmed in larger prospective studies.

Serum TMAO originates from dietary trimethylamine and the metabolism of compounds such as carnitine, betaine, and choline, which are abundant in animal-based foods. The gut microbiota converts these substrates into trimethylamine (TMA), which is subsequently oxidized in the liver by flavin-containing monooxygenase (FMO) to form TMAO. TMAO is now recognized as an important prognostic biomarker for cardiovascular diseases, reflecting the intricate interplay between diet, gut microbial metabolism, and cardiometabolic health [16,19]. In a systematic review and meta-analysis including 25 studies, Lombardo et al. [16] demonstrated that plant-based dietary patterns—such as the Mediterranean, vegetarian, and vegan diets—were effective in reducing TMAO concentrations, whereas animal-based diets, including the Paleo diet, high-meat diets, and omnivorous patterns, were associated with higher serum TMAO levels. Similarly, in a randomized, double-blind pilot study examining the impact of fiber supplementation on intestinal TMAO formation following beef consumption, fiber supplementation significantly attenuated TMAO production, particularly among individuals with lower habitual meat intake (<3 times per week; *p* = 0.029, 95% CI) [18]. Consistent with these findings, in the present study, individuals with lower TMAO concentrations had significantly higher intakes of total dietary fiber (*p* = 0.004), soluble fiber (*p* = 0.029), and insoluble fiber (*p* = 0.004) compared with those with higher TMAO levels. Moreover, plant protein intake was slightly but significantly higher among participants with lower TMAO levels (*p* = 0.040), while no significant differences were observed for carbohydrate, total protein, total fat, specific fatty acid, cholesterol intake, or Mediterranean diet adherence score (*p* > 0.05 for all). Taken together, these findings suggest that higher fiber and plant protein intake may be associated with lower circulating TMAO concentrations; however, whether these associations translate into improved cardiometabolic health requires confirmation in prospective and interventional studies. Within the PREDIMED study, which involved individuals with high cardiovascular risk, Hernández-Alonso et al. [33] found that C20:4 carnitine was positively associated with plant protein intake but negatively associated with animal protein, indicating that the source of dietary protein may differentially influence circulating carnitine and acylcarnitine metabolism. Unlike these findings, the present study also found that plant protein intake was slightly lower among participants with higher serum L-carnitine concentrations (*p* = 0.037). Elsewhere, recent human evidence from the African-PREDICT observational longitudinal study demonstrated that higher dietary fiber intake was associated with lower serum free and short-chain acylcarnitine levels, indicating an inverse relationship between fiber intake and circulating carnitine metabolites [34]. In contrast, in the present study, participants with higher L-carnitine concentrations exhibited greater total dietary fiber intake (*p* = 0.008), including both soluble (*p* = 0.018) and insoluble fiber (*p* = 0.013), compared with those with lower L-carnitine levels. This finding may reflect differences in metabolic context or compensatory mechanisms influencing carnitine metabolism among individuals with type 2 diabetes.

Gut microbiota-derived metabolites, particularly trimethylamine *N*-oxide (TMAO), have been implicated in the development of metabolic and cardiovascular diseases. Elevated serum TMAO levels are linked to oxidative stress, endothelial dysfunction, and impaired glucose metabolism, contributing to vascular complications in T2DM. However, this association may not be entirely causal, as renal impairment and gut dysbiosis in diabetes can elevate TMAO levels, suggesting its role as both a biomarker and mediator of disease progression [12]. In a Dutch cohort study, higher circulating TMAO concentrations were independently associated with an increased risk of cardiovascular mortality in individuals with type 2 diabetes. Over a 10-year follow-up period, participants in the highest TMAO tertile had nearly twice the risk of cardiovascular death compared with those in the lowest tertile (adjusted HR = 1.93; 95% CI: 1.11–3.34; *p* = 0.02) [10]. In another single-center cohort study, higher serum TMAO levels independently predicted major adverse cardiovascular events (MACEs) in patients with T2DM. Over a median 7-year follow-up of 1463 participants, those in the highest TMAO quartile had a 32% higher risk of MACE (HR = 1.32; 95% CI: 1.02–1.70; *p* = 0.033) compared with those in lower quartiles [9]. Consistent with previous prospective evidence, participants with macrovascular complications in the present study had significantly higher serum TMAO concentrations than those without complications (*p* < 0.05), indicating a cross-sectional association between circulating TMAO concentrations and macrovascular complication status in adults with T2DM.

L-carnitine, the active form of dietary carnitine and a key precursor of TMAO, has drawn increasing attention for its dual role in cardiovascular health: while essential for fatty acid oxidation and cardiac energy metabolism, its metabolism to the proatherogenic compound TMAO has linked it to increased cardiovascular risk [13]. In a prospective nested case–control study, a greater longitudinal increase in serum L-carnitine was associated with a higher risk of coronary heart disease, independent of baseline levels (RR = 1.36; 95% CI: 0.999–1.84 per 1-SD increase) [11]. Conversely, in a pooled analysis of two long-term Danish cohort studies involving individuals with type 2 diabetes and albuminuria followed for up to 21.9 years, serum L-carnitine concentrations were not associated with cardiovascular disease risk, suggesting that circulating carnitine levels alone may not independently predict cardiovascular outcomes in this high-risk population [15]. In the present study, conducted among adults with T2DM and preserved renal function, serum L-carnitine concentrations were significantly higher in participants with macrovascular complications than in those without (*p* < 0.05), indicating a cross-sectional association that warrants further investigation in prospective studies.

Food insecurity reflects an inability to obtain sufficient and nutritious food and is consistently linked to poor physical and mental health, lower academic and economic achievement, and an elevated risk of obesity, diabetes, and other chronic diseases [35]. In a cross-sectional study conducted among American adults, food insecurity was found to be more prevalent among individuals with type 2 diabetes, with approximately 16% of adults with diabetes reporting food insecurity compared to 9% of those without diabetes, and the prevalence was even higher among those with more advanced disease—19% among insulin users and 22% among individuals with diabetic eye or kidney complications [36]. With a substantially higher prevalence than previously reported, a case–control study conducted in Iran found that food insecurity affected 66.7% of individuals with type 2 diabetes compared with 41.5% of controls, highlighting the significant burden of food insecurity among diabetic populations in developing countries [37]. In Türkiye, food insecurity among individuals with type 2 diabetes had not been previously assessed, so this study set out to address this gap. According to our findings, only 16.7% of participants were food-secure, whereas 34.2% experienced mild food insecurity, 45.8% moderate food insecurity, and 3.3% severe food insecurity, revealing that almost half of the participants with T2DM lived with moderate-to-severe food insecurity.

Despite growing evidence linking food insecurity to adverse dietary patterns and cardiometabolic risk [35,38], no human study before ours directly examined whether circulating TMAO or L-carnitine concentrations differ across levels of food insecurity. Thus, it is interesting to note that, in the present study, participants experiencing moderate or severe food insecurity demonstrated significantly lower serum TMAO (*p* < 0.01) and L-carnitine concentrations (*p* < 0.05) compared with those reporting no or mild food insecurity. By identifying significantly lower TMAO and L-carnitine concentrations among food-insecure adults with type 2 diabetes, this study provides novel observational evidence that food insecurity is associated with differences in circulating gut-derived metabolites. However, because gut microbiota composition was not directly assessed, the mechanisms underlying these associations remain speculative. Future studies integrating dietary assessment, microbiome profiling, and metabolomics are needed to clarify the biological pathways involved. Differences in dietary access, food choices, intake of precursor nutrients, and eating patterns may offer plausible explanations for the observed variation in circulating TMAO and L-carnitine concentrations; however, these potential pathways were not directly evaluated in the present study and therefore remain speculative.

Overall, this study integrates several methodological strengths that enhance its scientific rigor: First, unlike most questionnaire-based studies, this research integrated objective biochemical biomarkers, specifically gut-derived metabolites such as TMAO and L-carnitine, providing a biochemical dimension to the analysis of their relationships with macrovascular complications, dietary patterns, and food insecurity in individuals with type 2 diabetes. Second, the study employed validated and widely used instruments—including the Food Insecurity Experience Scale (FIES), Mediterranean Diet Adherence (MDA) Scale, and International Physical Activity Questionnaire (IPAQ)—to support comparability of psychosocial and lifestyle assessments. Third, the statistical approach extended beyond descriptive comparisons: multivariable models incorporated clinically relevant covariates identified a priori, multinomial logistic regression was used as the primary approach for the mutually exclusive three-level HbA1c outcome, and separate binary models were retained as sensitivity analyses to examine the robustness of the findings across alternative model specifications. Together with the integrated assessment of dietary quality, food insecurity, gut-derived metabolites, and clinical outcomes, this multidimensional analytical framework represents a notable strength of the study and provides a more comprehensive perspective on their complex cross-sectional interrelationships in adults with T2DM. Nevertheless, these methodological strengths should be considered alongside the inherent limitations of the cross-sectional design, modest sample size, and limited precision of subgroup estimates.

Several limitations of this study should therefore be acknowledged. First, the cross-sectional design precludes causal inference between food insecurity, dietary adherence, and metabolic outcomes. Second, the single-center setting, moderate sample size, and predominance of female participants may limit the generalizability of the findings to broader and more diverse populations with T2DM. Third, dietary intake data were based on self-reported 24-h recalls, which are subject to recall bias and underreporting, particularly among individuals with obesity or food insecurity. Fourth, although validated tools were used, biomarker measurements were limited to serum TMAO and L-carnitine, without inclusion of other gut-derived metabolites or microbiota composition data, which could provide deeper mechanistic insights. Fifth, although clinically relevant covariates were selected a priori and model diagnostics supported the adequacy of the fitted models, the modest sample size relative to the number of exposure categories resulted in wide confidence intervals for several subgroup-specific estimates, limiting the precision of these findings. Sixth, HbA1c categories were derived from tertiles of the study population (n = 40 per tertile) rather than established clinical thresholds. Although this approach ensured balanced subgroup sizes for the regression analyses, it may limit the direct clinical interpretability and generalizability of the findings. Lastly, because multiple hypothesis tests were performed, statistically significant results should be interpreted alongside the reported effect sizes and confidence intervals rather than *p*-values alone. Collectively, these considerations indicate that the present findings should be regarded as exploratory and warrant confirmation in larger prospective studies.

## 5. Conclusions

In conclusion, this cross-sectional study identified exploratory relationships among food insecurity, Mediterranean diet adherence, gut-derived metabolites, glycemic control, and macrovascular complications in adults with type 2 diabetes mellitus. In the primary multinomial analysis, combined food insecurity–MDA patterns were not significantly associated with HbA1c tertile membership after multivariable adjustment, although some subgroup estimates suggested possible associations and were imprecise. Serum TMAO and L-carnitine concentrations differed according to macrovascular complication and food insecurity status and were also associated with selected dietary components. The observed relationships between dietary fiber, plant protein, and circulating metabolites should be interpreted as cross-sectional associations rather than evidence of microbiota-mediated modulation, particularly because gut microbiota composition and function were not directly assessed. Overall, these hypothesis-generating findings highlight potentially relevant links among food security, dietary factors, and gut-derived metabolites in T2DM and provide a rationale for larger prospective studies incorporating repeated dietary assessment and direct characterization of microbiota-related pathways.

## Figures and Tables

**Table 3:** Adjusted means of macronutrient intake and MDA score according to serum TMAO concentration among patients with T2DM.

	Serum TMAO Concentration				
	<1.29 ng/mL (n = 58)	≥1.29 ng/mL (n = 61)	F_adj_	p_adj_	Partial η^2^
**Carbohydrate (g/day)**					
Crude Mean ± SD	133.41 ± 55.44	121.09 ± 47.79			
Adjusted Mean ± SE	131.98 ± 4.04	124.24 ± 3.94	1.117	0.331	0.020
**Dietary fiber (g/day)**					
Crude Mean ± SD	14.69 ± 6.49	14.29 ± 7.05			
Adjusted Mean ± SE	15.21 ± 0.69	13.95 ± 0.67	5.691	**0.004**	0.095
**Soluble fiber (g/day)**					
Crude Mean ± SD	4.53 ± 2.09	4.70 ± 2.78			
Adjusted Mean ± SE	4.74 ± 0.27	4.55 ± 0.27	3.657	**0.029**	0.063
**Insoluble fiber (g/day)**					
Crude Mean ± SD	9.90 ± 4.92	9.21 ± 4.40			
Adjusted Mean ± SE	10.21 ± 0.51	9.01 ± 0.50	5.928	**0.004**	0.098
**Protein (g/day)**					
Crude Mean ± SD	47.40 ± 27.87	48.95 ± 17.01			
Adjusted Mean ± SE	47.50 ± 1.66	49.49 ± 1.62	0.596	0.553	0.011
**Plant protein (g/day)**					
Crude Mean ± SD	21.24 ± 11.08	20.60 ± 9.78			
Adjusted Mean ± SE	21.32 ± 0.99	20.81 ± 0.97	3.304	**0.040**	0.057
**Total fat (g/day)**					
Crude Mean ± SD	46.35 ± 28.61	45.44 ± 19.78			
Adjusted Mean ± SE	45.20 ± 1.71	47.43 ± 1.67	0.941	0.394	0.017
**Saturated fatty acids (g/day)**					
Crude Mean ± SD	17.09 ± 11.50	18.16 ± 10.22			
Adjusted Mean ± SE	16.51 ± 0.91	19.09 ± 0.89	2.144	0.122	0.038
**Monounsaturated fatty acids (g/day)**					
Crude Mean ± SD	16.63 ± 11.27	15.78 ± 7.32			
Adjusted Mean ± SE	16.36 ± 0.78	16.35 ± 0.77	0.588	0.557	0.011
**Polyunsaturated fatty acids (g/day)**					
Crude Mean ± SD	8.69 ± 6.46	7.63 ± 4.21			
Adjusted Mean ± SE	8.50 ± 0.55	7.95 ± 0.54	0.785	0.459	0.014
**Cholesterol (mg/day)**					
Crude Mean ± SD	251.7 ± 173.8	261.9 ± 145.9			
Adjusted Mean ± SE	245.7 ± 18.2	271.2 ± 17.8	1.107	0.334	0.020
**MDA score**					
Crude Mean ± SD	6.83 ± 2.50	6.08 ± 2.30			
Adjusted Mean ± SE	6.77 ± 0.32	6.12 ± 0.31	1.063	0.349	0.019

Boldface *p*-values indicate statistical significance at *p* < 0.05. Values are presented as mean ± standard deviation (crude) and adjusted mean ± standard error (ANCOVA-adjusted for covariates). Crude means are presented for descriptive purposes only; F_adj_ and p_adj_ values are based on ANCOVA models adjusted for age, duration of type 2 diabetes mellitus, gender, antidiabetic medication use, insulin therapy, total physical activity level, and energy intake. Participants are classified according to median serum TMAO concentration.

**Table 4:** Adjusted means of macronutrient intake and MDA score according to serum L-carnitine concentration among patients with T2DM.

	Serum L-Carnitine Concentration				
	<1.14 ng/mL (n = 61)	≥1.14 ng/mL (n = 58)	F_adj_	p_adj_	Partial η^2^
**Carbohydrate (g/day)**					
Crude Mean ± SD	131.8 ± 53.4	122.1 ± 50.0			
Adjusted Mean ± SE	132.4 ± 3.9	123.5 ± 4.0	1.419	0.246	0.025
**Dietary fiber (g/day)**					
Crude Mean ± SD	13.89 ± 6.00	15.11 ± 7.48			
Adjusted Mean ± SE	14.27 ± 0.67	14.88 ± 0.69	5.001	**0.008**	0.084
**Soluble fiber (g/day)**					
Crude Mean ± SD	4.32 ± 2.03	4.93 ± 2.82			
Adjusted Mean ± SE	4.44 ± 0.26	4.86 ± 0.27	4.165	**0.018**	0.071
**Insoluble fiber (g/day)**					
Crude Mean ± SD	9.22 ± 4.31	9.89 ± 5.00			
Adjusted Mean ± SE	9.48 ± 0.50	9.72 ± 0.51	4.504	**0.013**	0.076
**Protein (g/day)**					
Crude Mean ± SD	47.28 ± 22.31	49.15 ± 17.41			
Adjusted Mean ± SE	48.00 ± 1.62	49.07 ± 1.66	0.343	0.711	0.006
**Plant protein (g/day)**					
Crude Mean ± SD	21.04 ± 10.90	20.75 ± 9.93			
Adjusted Mean ± SE	21.45 ± 0.96	20.65 ± 0.99	3.404	**0.037**	0.059
**Total fat (g/day)**					
Crude Mean ± SD	45.35 ± 27.64	46.45 ± 20.64			
Adjusted Mean ± SE	44.71 ± 1.66	48.05 ± 1.70	1.477	0.233	0.026
**Saturated fatty acids (g/day)**					
Crude Mean ± SD	16.91 ± 11.31	18.41 ± 10.36			
Adjusted Mean ± SE	16.57 ± 0.89	19.16 ± 0.91	2.173	0.119	0.038
**Monounsaturated fatty acids (g/day)**					
Crude Mean ± SD	16.37 ± 10.91	16.01 ± 7.64			
Adjusted Mean ± SE	16.26 ± 0.76	16.46 ± 0.78	0.605	0.548	0.011
**Polyunsaturated fatty acids (g/day)**					
Crude Mean ± SD	8.29 ± 6.25	7.99 ± 4.45			
Adjusted Mean ± SE	8.18 ± 0.54	8.26 ± 0.55	0.545	0.581	0.010
**Cholesterol (mg/day)**					
Crude Mean ± SD	247.7 ± 176.7	266.6 ± 140.2			
Adjusted Mean ± SE	244.6 ± 17.7	273.6 ± 18.2	1.255	0.289	0.023
**MDA score**					
Crude Mean ± SD	6.52 ± 2.51	6.36 ± 2.34			
Adjusted Mean ± SE	6.45 ± 0.31	6.42 ± 0.32	0.046	0.955	0.001

Boldface *p*-values indicate statistical significance at *p* < 0.05. Values are presented as mean ± standard deviation (crude) and adjusted mean ± standard error (ANCOVA-adjusted for covariates). Crude means are presented for descriptive purposes only; F_adj_ and p_adj_ values are based on ANCOVA models adjusted for age, duration of type 2 diabetes mellitus, gender, antidiabetic medication use, insulin therapy, total physical activity level, and energy intake. Participants are classified according to median serum L-carnitine concentration.

## Data Availability

The data presented in this study are not publicly available due to ethical and privacy restrictions related to participant confidentiality and the conditions of informed consent approved by the institutional ethics committee. Anonymized data may be made available by the corresponding author upon reasonable request and, where applicable, subject to approval by the relevant ethics committee.

## Supplementary Materials

The following supporting information can be downloaded at: https://www.mdpi.com/article/10.3390/nu18162695/s1, Table S1. Sensitivity analysis using separate multivariable binary logistic regression models across HbA1c categories (reference exposure: FI−/MDA↑); Table S2. Assessment of multicollinearity among covariates included in the adjusted regression models; Table S3. Model diagnostics for the adjusted multivariable binary logistic regression models; Table S4. Adjusted pairwise comparisons corresponding to Figure 1.
