# Supplementary material for: A Cross-Sectional Study of Food Insecurity, Mediterranean Diet Adherence, and Gut-Derived Metabolites and Their Associations with Glycemic Control and Macrovascular Complications in Adults with Type 2 Diabetes
Source: Nutrients. 2026 Aug 18;18(16):2695. doi: 10.3390/nu18162695 (PMC13516299; doi:10.3390/nu18162695)
Supplement: Supplementary file 1 [file nutrients-18-02695-s001.zip › nutrients-4472882-supplementary.pdf]

Supplementary Table S1. Sensitivity analysis using separate multivariable binary logistic regression models across HbA1c categories (reference exposure: FI−/MDA↑)

Pattern of food insecurity × diet quality	HbA1c <6.62 AOR [95% CI]	p	HbA1c 6.62–8.22 AOR [95% CI]	p	HbA1c >8.22 AOR [95% CI]	p
**FI− / MDA↓**	0.42 (0.16–1.10)	0.078	1.36 (0.40–4.61)	0.624	0.94 (0.34–2.62)	0.909
**FI+ / MDA↓**	1.51 (0.41–5.58)	0.534	3.80 (0.92–15.8)	0.066	3.09 (0.98–9.73)	0.054
**FI+ / MDA↑**	1.06 (0.34–2.62)	0.909	5.18 (1.40–19.2)	0.014	4.32 (1.39–13.4)	0.012
**FI− / MDA↑**	Reference	—	Reference	—	Reference	—

FI: Food insecurity status (− secure, + insecure); MDA: adherence to Mediterranean diet (↓ low adherence, ↑ high adherence). Each binary model compared the indicated HbA1c category with the remaining two categories combined. AORs are adjusted for age, duration of type 2 diabetes mellitus, gender, body mass index, total physical activity level, oral antidiabetic medication use, insulin therapy, and total energy intake. HbA1c categories were derived from tertiles of the study population and do not represent established clinical thresholds.

Supplementary Table S2. Assessment of multicollinearity among covariates included in the adjusted regression models

Variable	Variance Inflation Factor (VIF)
Age	1.349
Type 2 diabetes duration	1.470
Sex	1.253
Body mass index (BMI)	1.207
Oral antidiabetic medication use	1.389
Insulin use	1.318
Physical activity	1.262
Total energy intake	1.082

Supplementary Table S3. Model diagnostics for the adjusted multivariable binary logistic regression models

Diagnostic	HbA1c <6.62	HbA1c 6.62–8.22	HbA1c >8.22
−2 Log likelihood	96.26	140.36	108.29
McFadden pseudo-R^2	0.370	0.081	0.291
Classification accuracy (%)	82.5	68.3	75.8
ROC AUC	0.878	0.698	0.845
Hosmer–Lemeshow χ^2	17.15	5.14	11.39
Hosmer–Lemeshow df	8	8	8
Hosmer–Lemeshow p-value	0.029	0.743	0.180
Minimum VIF	1.08	1.08	1.08
Maximum VIF	1.47	1.47	1.47

Supplementary Table S4. Adjusted pairwise comparisons corresponding to Figure 1

Outcome	Comparison	n	Adjusted mean (95% CI)	Adjusted mean difference (95% CI)	Partial η^2	Exact p-value
	**Macrovascular Complications**					
TMAO	Absent	82	1.39±0.05 (1.29-1.50)	-0.231 (-0.433 to -0.029)	0.044	**0.026***
	Present	37	1.63±0.08 (1.46-1.79)			
L-carnitine	Absent	82	1.25±0.06 (1.13-1.36)	-0.233 (-0.454 to -0.012)	0.039	**0.039***
	Present	37	1.48±0.09 (1.30-1.66)			
	**Food Insecurity Status**					
TMAO	No or mild	60	1.58±0.06 (1.46-1.70)	0.232 (0.059-0.406)	0.061	**0.009****
	Moderate or severe	59	1.35±0.06 (1.23-1.47)			
L-carnitine	No or mild	60	1.43±0.06 (1.30-1.56)	0.222 (0.038-0.406)	0.050	**0.018***
	Moderate or severe	59	1.21±0.07 (1.08-1.34)			

Values are adjusted estimated marginal means derived from ANCOVA after adjustment for age, sex, duration of diabetes, body mass index, insulin use, oral antidiabetic medication, physical activity, and total energy intake. Adjusted mean differences were calculated as Group 1 minus Group 2 (Group 1 − Group 2). Positive values indicate higher adjusted means in Group 1, whereas negative values indicate higher adjusted means in Group 2. Exact p-values correspond to the ANCOVA group comparison. Partial η^2 is presented as the measure of effect size. Asterisks indicate statistical significance (*$p < 0.05$, **$p < 0.01$).
